# Sex-dependent diversity in ventral tegmental dopaminergic neurons and developmental programing: A molecular, cellular and behavioral analysis

**DOI:** 10.1016/j.neuroscience.2014.05.033

**Published:** 2014-12-12

**Authors:** G.E. Gillies, K. Virdee, S. McArthur, J.W. Dalley

**Affiliations:** a Division of Brain Sciences, Imperial College London, Hammersmith Hospital, London, UK; b Behavioural and Clinical Neuroscience Institute, University of Cambridge, Downing Street, Cambridge CB2 3EB, UK; c Department of Psychology, University of Cambridge, Downing Street, Cambridge CB2 3EB, UK; d William Harvey Research Institute, Barts and The London School of Medicine and Dentistry, Charterhouse Square, London EC1 6BQ, UK; e Department of Psychiatry, University of Cambridge, Addenbrooke’s Hospital, Hill’s Road, Cambridge CB2 2QQ, UK

**Keywords:** AGT, antenatal glucocorticoid treatment, AR, androgen receptor, DA, dopamine, DAT, DA transporter, DHT, dihydrotestosterone, ER, estrogen receptor, HPA, hypothalamo–pituitary–adrenal, IR, immunoreactivity, mPFC, medial prefrontal cortex, NAc, nucleus accumbens, NSDA, nigrostriatal dopaminergic, SNc, substantia nigra pars compacta, TH-IR, tyrosine hydroxylase immunoreactivity, VTA, ventral tegmental area, glucocorticoids, nucleus accumbens, prefrontal cortex, addiction, stimulants, stress

## Abstract

The knowledge that diverse populations of dopaminergic neurons within the ventral tegmental area (VTA) can be distinguished in terms of their molecular, electrophysiological and functional properties, as well as their differential projections to cortical and subcortical regions has significance for key brain functions, such as the regulation of motivation, working memory and sensorimotor control. Almost without exception, this understanding has evolved from landmark studies performed in the male sex. However, converging evidence from both clinical and pre-clinical studies illustrates that the structure and functioning of the VTA dopaminergic systems are intrinsically different in males and females. This may be driven by sex differences in the hormonal environment during adulthood (‘activational’ effects) and development (perinatal and/or pubertal ‘organizational’ effects), as well as genetic factors, especially the *SRY* gene on the Y chromosome in males, which is expressed in a sub-population of adult midbrain dopaminergic neurons. Stress and stress hormones, especially glucocorticoids, are important factors which interact with the VTA dopaminergic systems in order to achieve behavioral adaptation and enable the individual to cope with environmental change. Here, also, there is male/female diversity not only during adulthood, but also in early life when neurobiological programing by stress or glucocorticoid exposure differentially impacts dopaminergic developmental trajectories in male and female brains. This may have enduring consequences for individual resilience or susceptibility to pathophysiological change induced by stressors in later life, with potential translational significance for sex bias commonly found in disorders involving dysfunction of the mesocorticolimbic dopaminergic systems. These findings highlight the urgent need for a better understanding of the sexual dimorphism in the VTA if we are to improve strategies for the prevention and treatment of debilitating conditions which differentially affect men and women in their prevalence and nature, including schizophrenia, attention/deficit hyperactivity disorder, autism spectrum disorders, anxiety, depression and addiction.

## Highlights

• Sex hormones and genomic factors underpin sex dimorphism in VTA structure and function.
• Environmental stressors differentially impact male and female VTA dopaminergic systems.
• Sex diversity in VTA has relevance for sex bias in dopaminergic dysfunction.

## Introduction

The last half of the twentieth century saw an exponential rise in the scientific evidence for sex differences in the brain. Yet, the implications of these findings were ignored, if not suppressed, largely due to the politically correct notion that claims for brain sex differences challenged equality and were retrogressive (Kimura, 1992, Moir and Jessel, 1992, McCarthy et al., 2012). However, it is increasingly apparent that neurological and neuropsychiatric disorders show significant sex differences in susceptibility, prevalence, presentation (symptoms), progression, pathology and response to treatments (Cahill, 2006, Cosgrove et al., 2007, Gillies and McArthur, 2010b, Ngun et al., 2011, Becker et al., 2012, McCarthy et al., 2012). This has led to the realization that different preventive, diagnostic and treatment approaches may be required for men and women, which, in turn, highlights the urgent need for a better understanding of specific pathways that exhibit biological sex differences in the brain, along with mechanisms which generate these differences (Health, 2011). The goal of this review is to consider sex, distinguished as being male or female according to reproductive organs and chromosomal complement (male XY and female XX sex chromosomes), as a factor in diversity of the ventral tegmental area (VTA), which impacts both the physiology and pathology of the dopaminergic systems originating in the VTA. We shall first review the evidence for biological sex dimorphisms in the normal dopaminergic network, followed by the key factors (sex hormones and chromosomes) which underpin these differences. We then provide a brief summary of sex differences in dopaminergic malfunction, and develop the hypothesis that a sexually dimorphic response of the mesocorticolimbic system to stressors and stress hormones, especially glucocorticoids, during adulthood or development represents a mechanism which may contribute to sex biases commonly found in dopamine (DA)-associated disorders.

## Sex differences in normal midbrain dopaminergic systems

Sex differences in the hypothalamic circuitry governing reproduction and mating behaviors are widely accepted as being fundamental to the survival of dioescious vertebrates. Mounting evidence now suggests that the midbrain dopaminergic systems are also sexually dimorphic. We have recently reviewed the molecular, cellular and functional sex differences in the nigrostriatal dopaminergic (NSDA) system, which originates in the midbrain region of the substantia nigra pars compacta (SNc) and projects to the dorsal striatum (Gillies and McArthur, 2010a, Gillies and McArthur, 2010b, McArthur and Gillies, 2011, Gillies et al., 2014). This regulates sensorimotor function, and its sexually dimorphic nature is thought to contribute to sex differences in the prevalence and nature of Parkinson’s disease, where NSDA degeneration is a defining pathology (Dexter and Jenner, 2013). Emerging evidence suggests that sex differences are also present in the dopaminergic pathways originating in the adjacent VTA. One sub-population of VTA dopaminergic neurons projects to the ventral striatum, especially the nucleus accumbens (NAc), to form the mesolimbic pathway, which is a key regulator of emotional social behaviors, reward-associated behaviors, motivation and feeding: the other sub-population projects to the prefrontal cortex to form the mesocortical pathway, which provides critical regulation of cognition, and working memory (Robbins, 2000, Bjorklund and Dunnett, 2007, Everitt et al., 2008, O’Connell and Hofmann, 2011). Studies in humans and experimental animals provide considerable evidence for sex differences in these behaviors, and the extent to which this may involve sex differences in the mesocorticolimbic system is considered in this section.

### Humans

Sex differences in specific aspects of motivation, reward cognition, behavior and affect, have been reviewed in detail elsewhere (Cahill and McGaugh, 1998, Sherwin, 2003, Cahill, 2006, Cosgrove et al., 2007, Guiso et al., 2008, Sherwin and Henry, 2008, Hines, 2011b, Becker et al., 2012). For example, women appear to be more sensitive than men to the rewarding effects of psychoactive drugs, suggesting differences in dopaminergic activity in the NAc as a potential contributory mechanism (Carroll et al., 2004, Lynch, 2006, Becker and Hu, 2008). Differences are also seen in prefrontal processes, such as working memory, involving the mesocortical system. These include sex differences in performance in tests of verbal memory and visuospatial memory as well as manual dexterity, which tend to show a female advantage (Schattmann and Sherwin, 2007a), whereas men generally outperform women in spatial tasks requiring mental re-orientation, as well as target-directed motor skills (Kimura and Harshman, 1984, Kimura, 1992, Hines, 2011a). In humans, sociocultural factors may contribute both to underlying differences in neurological development as well as cognitive functions. However, even when sociocultural differences have been accounted for, sex differences in brain function may persist (Guiso et al., 2008), and human behavioral sex differences also have parallels in non-human mammalian species that lack identical sociocultural pressures. Together, such observations argue for intrinsic factors as being primary drivers of brain sex differences. Recent data from real-time *in vivo* neuroimaging studies provide key, direct evidence that fundamental sex differences in the midbrain dopaminergic systems contribute to behavioral dimorphisms. For example, although there appears to be little sex difference in striatal DA release at rest, women were found to have greater pre-synaptic DA synthetic capacity, striatal DA transporter (DAT) density and accumulation of the DA precursor radionuclide, F-Fluorodopa (FDOPA), compared with men (Lavalaye et al., 2000, Mozley et al., 2001, Laakso et al., 2002), whereas the affinity of the D2 receptor subtype was greater in men (Pohjalainen et al., 1998). In healthy young subjects performing behavioral tests associated with dopaminergic activity, neuroimaging studies found a relationship between striatal DA availability and executive and motor functioning in women, but not in men (Mozley et al., 2001). Significant sex differences were also found when correlating changes in cognition and affect with DA release in striatal and extra-striatal regions after amphetamine administration (Riccardi et al., 2011). Functional neuroimaging studies have also linked sex differences in reward-related behaviors to sex dimorphisms in the mesolimbic dopaminergic response, which were reported to be greater in men than women (Munro et al., 2006, Diekhof et al., 2012). Although investigations of this nature are in their infancy, these observations suggest that there are intrinsic sex differences in basal DA neuron dynamics, and that cognitive and motor functions may be differentially regulated by midbrain DA systems in men and women. This provides support for the concept that male and female brains may be reliant on different strategies to reach the same goal, and that similar performance between the sexes does not necessitate the same neural mechanisms and information processing (Cahill, 2006).

### Pre-clinical studies

**Fig. 1:**
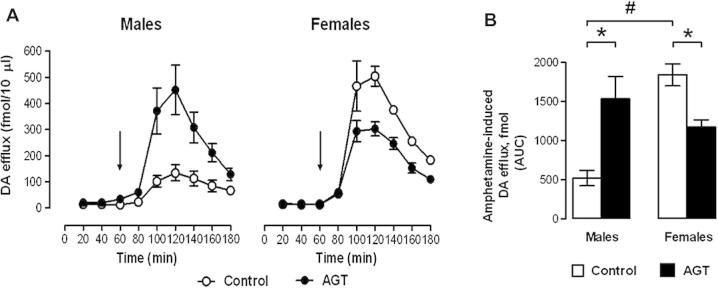
Sex differences in accumbal dopamine (DA) responses to amphetamine and impact of antenatal glucocorticoid treatment (AGT). Male and female rats exposed to AGT (dexamethasone, 0.5 μg/ml, in drinking water on gestational days 16–19; closed symbols in the line plots and solid bars in the bar plots) and controls (dams received normal drinking water; open symbols in the line plots and open bars in the bar plots) were tested in adulthood. Extracellular levels of DA in the NAc were assessed by *in vivo* microdialysis coupled with electrochemical detection. Microdialysis samples were collected every 20 min for 3 h and after the first three fractions (baseline) amphetamine (0.8 mg/kg *i.p*.) was administered. The line plots (A) depict DA levels in each 20-min fraction and the arrow indicates the point of amphetamine administration. The bar plot (B) shows cumulative DA release above baseline (area under the curve, AUC). Data were used only from those animals where placement of the dialysis probe in the core of the nucleus accumbens was confirmed post-mortem; statistical analyses were adjusted accordingly for differences in group sizes. *Controls:* Baseline levels of DA efflux were similar in males and females (A), whereas amphetamine-stimulated DA efflux was almost fourfold greater in females compared with males (A, B). *AGT:* Baseline levels of DA efflux were unaffected by AGT, whereas amphetamine-stimulated efflux was increased in males, but decreased in females compared with controls. Values represent means ± s.e.m for control males (*n* = 5), AGT males (*n* = 6), control females (*n* = 4) and AGT females (*n* = 5). ^#^*p* < 0.05, indicating a significant sex difference; ^∗^*p* < 0.05 indicating a significant effect of AGT. For full details see Virdee et al. (2013).

Investigations performed largely in rodents support, extend, and even predict, the human data. For example, investigations in gonad-intact rats using *in vivo* microdialysis (Fig. 1A) (Virdee et al., 2013) or voltammetry (Walker et al., 2000, Walker et al., 2006), report no sex differences in DA efflux in the NAc and striatum at rest, and striatal DA content is similar in male and female striata (Murray et al., 2003, Gillies et al., 2004, McArthur et al., 2005, Kuhn et al., 2010). However, these similarities appear to be achieved by a different balance in dopaminergic regulatory mechanisms, with a faster rate of uptake and release in females (Walker et al., 2006). Additionally, far greater extracellular levels of DA are found in females compared with males treated with the indirectly acting DA receptor agonists, amphetamine (Fig. 1) (Virdee et al., 2013) or cocaine (Walker et al., 2006), which both target DAT in the DA nerve terminals. Sex differences in mesolimbic activity are also confirmed using the typical antipsychotic, haloperidol. This drug enhances electrically stimulated DA signaling by blocking presynaptic, auto-inhibitory D2 receptors which inhibit DA release and stimulate uptake (Wu et al., 2002), and has a significantly greater effect on the overflow of DA into the synapse in females compared with males (Walker et al., 2006). Together, these observations suggest that the sex dimorphisms of the mesolimbic dopaminergic system could explain why psychoactive drugs, such as amphetamine and cocaine, induce greater locomotor responses and behavioral sensitization in female compared with male rats (Bowman and Kuhn, 1996, Becker et al., 2001, Walker et al., 2001), and why females show a greater sensitivity to haloperidol-induced catalepsy (Campbell et al., 1988).

**Fig. 2:**
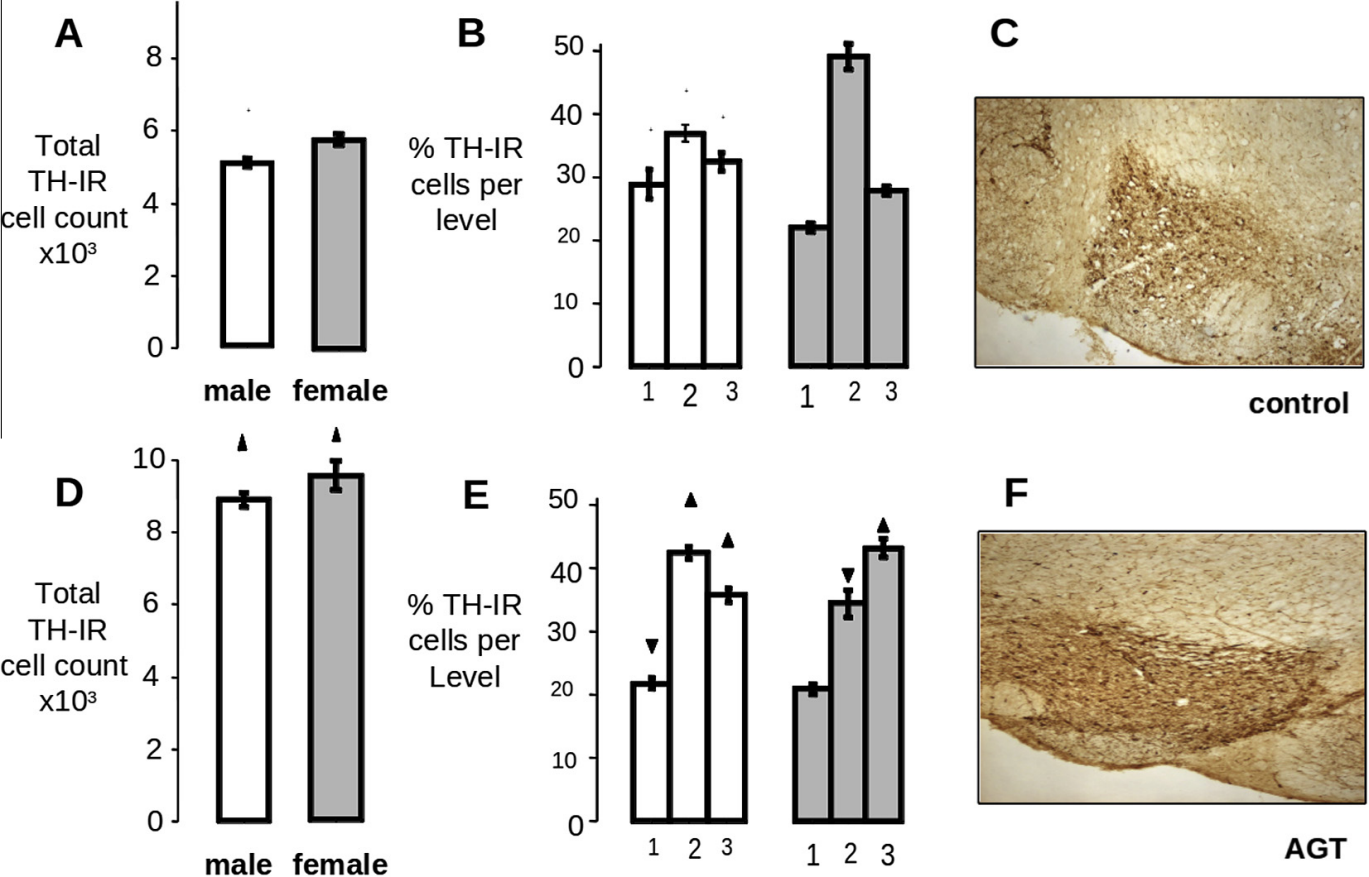
Sex differences and impact of antenatal glucocorticoid treatment (AGT) on the total cell count and distribution of tyrosine hydroxylase-immunoreactive (TH-IR) cells in the adult rat VTA. Brain slices containing the VTA from adult male and female rats exposed to AGT (dexamethasone, 0.5 μg/ml, in drinking water on gestational days 16–19) and controls (dams received normal drinking water) were processed for immunocytochemical detection of TH. The VTA was delineated by TH-IR and anatomical landmarks. In order to detect any regional differences throughout the nucleus, coronal sections containing the VTA were divided into three levels, each spanning 300 μm, beginning at −5.1 mm (level 1), −5.4 mm (level 2) and −5.7 mm (level 3) with respect to bregma. *Controls* (A–C): Total TH-IR cell counts were significantly greater in females (A). The distribution of these cells was relatively uniform throughout the three levels in males, whereas a greater proportion was found at level 2 in females. *AGT* (D–F): After AGT the total adult TH-IR cell counts were markedly increased in males and females, and their distribution across levels 1–3 was altered, which can be described as a rostro-caudal shift in cell number. ▴,▾, indicates significant effect of treatment, *p* < 0.05 increased or decreased respectively for dexamethasone treated *vs.* control animals; ^+^ indicates significant sex difference *p* < 0.05 vs*.* females in the same treatment group. For full details see McArthur et al., 2005, McArthur et al., 2007a.

Sex differences are also seen in performance in cognitive tasks, such as working memory, which measure prefrontal cortical processes (van Haaren et al., 1990, Ceccarelli et al., 2001). To a certain extent, these may reflect neuroanatomical sex differences which have been identified in the rat mesocortical dopaminergic system. For example, retrograde labeling studies to identify the VTA DA neurons projecting to the prelimbic area of the prefrontal cortex, the primary motor cortex and the premotor cortex, revealed large sex differences in the proportions of DA neurons making up all three mesocortical pathways (Kritzer and Creutz, 2008). For all three regions the proportion of back-labeled cells in females was approximately double than that seen in males. Structural sex differences have also been reported in the VTA, with females having a significantly greater number of DA perikarya (Fig. 2A), as well as a greater volume of this region, compared with males (McArthur et al., 2007a). In addition, the overall shape of the VTA, as delineated by the volumes occupied by the DA perikarya at different levels through the rostro-caudal axis of the nucleus, as well as the distribution of the DA neurons at each level, is sexually dimorphic, although the size of the individual perikarya is similar between the sexes (McArthur et al., 2007a) (Fig 2B). Overall, these cytoarchitectural differences can be described as a rostro-caudal shift in the volume and distribution of DA cells in females relative to males. As the VTA comprises sub-sets of neurons with region-specific input and output systems to govern its multi-functionality (Bjorklund and Dunnett, 2007, Roeper, 2013), these topographical differences may indicate altered patterns of connectivity which could underpin the neurochemical and behavioral sex differences.

Notably, the landmark studies on which we base our understanding of the molecular, electrophysiological, anatomical and functional diversity in the VTA (Roeper, 2013) have been carried out almost without exception (Fiorillo et al., 2008) in males. The foregoing discussion, therefore, highlights that more studies are required to understand the sexual diversity of the VTA.

## Mechanisms underlying sexual differentiation of the VTA dopaminergic systems

**Fig. 3:**
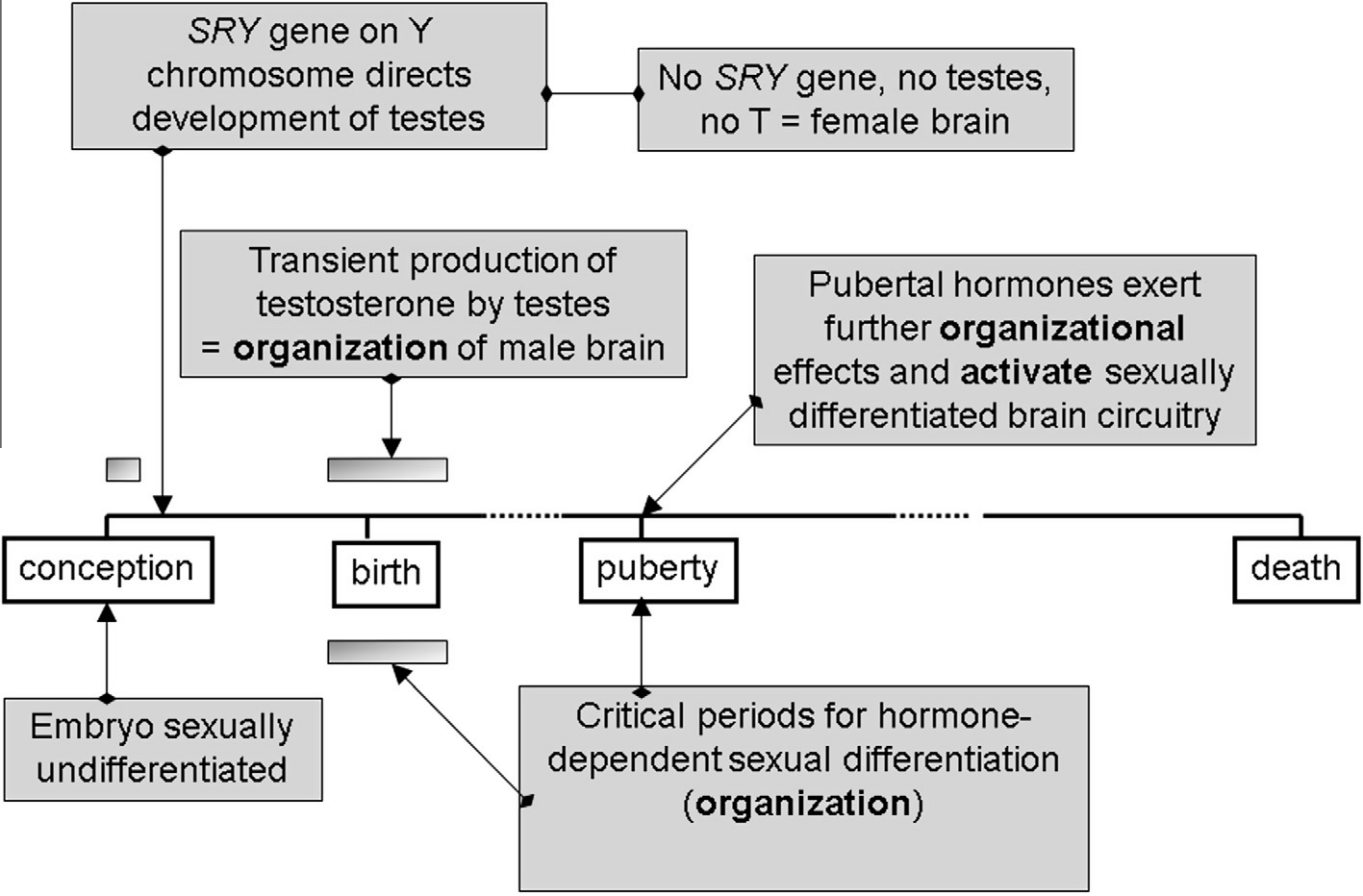
Schematic representation of hormone-dependent sexual differentiation of the brain. Early in gestation the *SRY* gene on the Y sex chromosome in males directs development of the testes. A transitory activation of the testes in males (but not the ovaries in females) during a critical developmental window (just before and after birth in rodents; approximately mid-gestation in human and non-human primates) means that the brain develops in a different hormonal environment in males and females, which establishes irreversible sex dimorphisms in specific neural circuits. Rising levels of gonadal steroids in the peripubertal period may exert further organizational effects (especially estradiol in females). From puberty onward, gonadal steroids activate the sexually dimorphic circuitry. This concept has arisen from extensive studies of hypothalamic circuitry controlling reproduction and reproductive behaviors, sexual differentiation of which is fundamental for survival of the species. Evidence suggests that extra-hypothalamic brain regions involved in learning and memory may also be subject to similar organizational and activational influences of gonadal steroids during development and adulthood, respectively (Luine et al., 1986, McEwen, 1999, Bangasser and Shors, 2008, Mitsushima et al., 2009), but how these principles apply specifically to the VTA dopaminergic system requires clarification.

Gonadal steroid hormones are major drivers of sex differences in the brain. It is well established that a transient perinatal rise in testosterone production by the developing testes is a principal factor in the masculinization/defeminization of the brain (Fig. 3) (Wilson and Davies, 2007, McCarthy, 2008, Arnold, 2009, McCarthy and Arnold, 2011). Limited evidence suggests that similar events prevail in the human fetus at a comparable stage of brain development, around the time of mid-gestation (Gillies and McArthur, 2010b). The processes which are affected include neurogenesis, neural migration, synaptogenesis, gliogenesis and programed cell death. In contrast to this early activation of the testes, ovarian steroidogenesis remains low at this stage of development. Therefore, during a critical window, the male and female brain develops in a very different hormonal environment, which leads to permanent differences in the hard-wiring of male and female brains. Recent evidence suggests that a second wave of irreversible, organizational influences occurs in late adolescence/puberty when the sex hormone environment begins to become sexually dimorphic once again with the rise of testosterone in males and estrogens and progesterone in females (Sisk and Zehr, 2005, Juraska et al., 2013). This stage involves neuronal and glial proliferation and morphological differentiation, as well as cell death in specific brain regions, and is associated with an active feminization of the brain, as distinct from the masculinizing/defeminizing processes during the perinatal period, although puberty may also include further masculinization. The post-pubertal hormonal environment then further augments the underlying dimorphisms in brain structure and function via activational, reversible influences of testosterone, estrogens and progesterone which differentially affect various aspects of neurotransmission in males and females, such as neurotransmitter synthesis, receptor expression and synaptic plasticity (McEwen, 1999, McEwen, 2002, McEwen and Alves, 1999, Pfaff, 2005). Although these concepts of hormone-directed sexual differentiation of the brain developed from studies of the central control of reproduction and reproductive behaviors by the hypothalamus, it is now recognized that they are more widely applicable in regions throughout the brain. In the following sections the specific relevance of these concepts to the VTA dopaminergic systems will be discussed, beginning with the activational influences in adulthood, which provide the bulk of the data. In addition, the dopaminergic systems have been instrumental in revealing that genetic factors, independent of sex hormones, also have a significant role to play in sexual differentiation of the VTA, which we summarize briefly.

### Sex hormone influences in adulthood

In humans and experimental species, many adult brain functions, such as cognitive abilities, attention, mood, reward and motivation, are influenced by the activational influences of gonadal factors in both sexes (van Haaren et al., 1990, McEwen, 1999, McEwen, 2002, McEwen, 2010, Sherwin, 2003, Daniel, 2006, Caldu and Dreher, 2007, Craig and Murphy, 2007, Luine, 2008, Brinton, 2009, Pike et al., 2009, Becker et al., 2012). As the VTA dopaminergic systems contribute to these functions to varying degrees, they are likely targets for hormone-dependent expression of sexual dimorphism.

#### The mesolimbic system

Women are more sensitive to the rewarding effects of psychoactive drugs than men (Carroll et al., 2004, Lynch, 2006, Becker and Hu, 2008), and the pattern of activation of reward circuitry in men differed from that seen in women during both the anticipation and delivery of rewards (Caldu and Dreher, 2007). In women the effects of psychoactive drugs also vary across the menstrual cycle (Carroll et al., 2004, Lynch, 2006, Becker and Hu, 2008), and functional magnetic resonance imaging studies reveal an increased responsiveness of the reward system to psychoactive drugs during the mid-follicular phase of the menstrual cycle, when estradiol levels are rising and relatively unopposed by progesterone (Caldu and Dreher, 2007). Overall the data identify estrogens in women as a driving factor for these differences, but the contribution of testosterone in men remains to be investigated systematically. Collectively, the human data suggest that gonadal steroids play an important role in driving sex differences in the adult mesolimbic dopaminergic system through classical, activational effects.

Animal studies confirm and extend the human studies and provide empirical support for the view that gonadal factors may be acting on a sexually differentiated mesolimbic dopaminergic circuitry (Becker, 1999, Gillies and McArthur, 2010b, Becker et al., 2012). For example, in female rats basal and amphetamine-stimulated concentrations of DA in the striatum (especially the NAc), as well as behavioral responses to amphetamine (locomotor activity and stereotypy), are positively correlated with endogenous estradiol levels as they fluctuate over the estrous cycle (Fink et al., 1996, Becker, 1999). Moreover, estradiol treatment reverses ovariectomy-induced attenuation of these neurochemical and behavioral responses (Xiao and Becker, 1994, Becker, 1999, Ohtani et al., 2001). In females estradiol also increases DA synthesis and turnover and markedly suppresses the density of striatal DAT, which critically regulates DA neuron dynamics (Pasqualini et al., 1995, McArthur et al., 2007b). In contrast, neither castration nor treatment of castrated rats with testosterone, or the non-aromatizable dihydrotestosterone (DHT; an androgen which, unlike testosterone cannot be metabolized to estradiol by endogenous aromatase enzymes) or estradiol had any significant effect on these neurological or behavioral parameters in males (Becker, 1999, McArthur et al., 2007b). Furthermore, the sexually dimorphic responses to estradiol would indicate an innate sexual differentiation of the underlying circuitry, most likely imprinted by factors early in development (see Section ‘Sex hormone influences during development’).

#### The mesocortical dopaminergic system

The mesocortical dopaminergic system and the prefrontal processes which it regulates, such as working memory, are also subject to the activational influences of circulating gonadal hormones in men and women (Hampson, 1990, Janowsky, 2006). However, there is no simple conclusion as to whether sex hormones facilitate, compromise or have no effect on learning and memory. Additionally, effects appear to be more complex than those in the mesolimbic system, with both estrogens and androgens reported to influence different aspects of cognition in women and men. In support of this, the brains of men and women express both androgen receptors (ARs) and estrogen receptors (ERs), with a similar, wide distribution between the sexes (Simerly et al., 1990, McEwen, 2002, Gillies and McArthur, 2010b). A peripheral source of androgens, such as dehydroepiandrosterone and androstenedione, comes from the adrenal glands in both sexes as well as the ovaries (Labrie et al., 2005), and in males estradiol can be synthesized from circulating testosterone by aromatase enzymes located in tissues throughout the body, including the brain (Simpson and Jones, 2006). In addition, the brain itself can be considered a steroidogenic organ, because it possesses the complement of steroid synthesizing enzymes, which enables synthesis of estrogens and androgens *de novo*, or by metabolism of peripherally derived precursors (Do Rego et al., 2009, Pelletier, 2010). These observations provide and infrastructure to explain why androgens in women (Miller et al., 2006) and estrogens in men (Cherrier et al., 2003) may influence mood and cognition, as well as vice-versa. They also provide the rationale for studying the effects of androgens and estrogens in both sexes.

Typically, human studies have investigated the effects of sex hormones on performance in memory tests which show sex differences. The data show, for example, that in young and older men DHT (a non-aromatizable androgen acting principally at ARs), but not estradiol, promoted performance in spatial visualization/memory tasks (typically male advantage) (Cherrier et al., 2003, Cherrier et al., 2005), whereas elevated levels of androgens in young women did not affect their performance in such tasks (Schattmann and Sherwin, 2007a, Schattmann and Sherwin, 2007b). This could suggest that the activational effects of androgens in females were not sufficient to masculinize the underlying circuitry, compatible with the view that the underlying circuitry is sexually dimorphic. In contrast, the performance of young men in tests typically showing a female advantage (verbal memory, visual memory) was promoted by testosterone only after aromatization to estradiol (Kampen and Sherwin, 1996, Cherrier et al., 2003). Positive effects of estrogens on cognition-related circuitry in the PFC and on performance in working memory tasks have also been confirmed in young women in a study combining positron emission tomography, a battery of neuropsychological tests and pharmacological manipulation of ovarian steroids, as well as in menopausal women with or without hormone replacement therapy (Berman et al., 1997, Keenan et al., 2001). In contrast, results of studies comparing normal, control young women and those with elevated testosterone levels due to polycystic ovarian syndrome suggest that testosterone compromises performance in cognitive tests showing a female advantage (verbal fluency, verbal memory, manual dexterity, visuospatial working memory), but has no effect on those showing a male advantage (mental rotation, spatial visualization, spatial perception, perceptual speed) (Schattmann and Sherwin, 2007a, Schattmann and Sherwin, 2007b). As permissible experimental designs in humans do not enable rigorous testing of hypotheses, it is difficult to draw clear conclusions from such studies, but they do indicate that sex hormones differentially affect human behavior and may be acting on a sexually differentiated circuitry (Gillies and McArthur, 2010b).

Similar to the human data, animal behavioral studies demonstrate that gonadal hormones influence performance in cognitive tests that are reliant on the PFC, although the effects of gonadectomy and treatment with estradiol or testosterone vary according to sex and test (Ceccarelli et al., 2001, Gibbs, 2005). Pre-clinical studies originally focused on the performance-enhancing effects of estradiol in ovariectomized female rats and rhesus monkeys, which have been correlated positively with effects on the growth of prefrontal spine synapses (Daniel, 2006, Wallace et al., 2006, Hajszan et al., 2008). However, subsequent work suggests that both estrogens and androgens may have positive effects on cognition and synapse remodeling in the PFC of both sexes (Hajszan et al., 2008). Studies looking specifically at the mesocortical dopaminergic system have been performed principally in male rodents. For example, castration increased the density of axons positive for tyrosine hydroxylase immunoreactivity (TH-IR) as well as extracellular resting DA levels in the male rat medial prefrontal cortex (mPFC), and this effect was reversed by treatment with testosterone, not estradiol (Kritzer, 2003, Kritzer and Creutz, 2008, Aubele and Kritzer, 2011). Castration also impaired DA-dependent meso-prefrontal functions such as operant spatial working memory, T-maze acquisition and novel object recognition acquisition in an androgen-, not estrogen-, dependent manner (Kritzer et al., 2007). These data are consistent with the human data where performance of certain prefrontal tasks in men has been correlated with circulating androgen, not estrogens (Cherrier et al., 2003). Castration of male rats has also been reported to cause a decline in prefrontal DA-dependent tests of motivation. Notably, however, this effect was reversed by treatment with estradiol, not testosterone (Kritzer et al., 2007). Yet other aspects of PFC function involving impulsivity and reversal learning were unaffected by castration or hormone treatment, and unrelated to PFC dopaminergic innervation (Kritzer and Creutz, 2008). These observations invite the speculation that sex steroid hormones differentially influence discrete VTA populations and their associated behaviors.

An androgenic rather than estrogenic influence on certain aspects of mPFC function in male rats is consistent with the finding that AR-immunoreactivity (IR), not ER-IR, was found to co-localize with TH-IR in a sub-set of VTA neurons which project to the prelimbic area of the mPFC (Kritzer and Creutz, 2008), indicating direct AR-mediated influences within dopaminergic neurons. In contrast, ERβ-IR predominated in the VTA dopaminergic neurons projecting to the primary motor cortex, where castration reduced TH-IR density in an estrogen-, not androgen-, dependent manner; in yet another set of dopaminergic neurons projecting to the premotor cortex, neither AR-IR nor ER-IR was detected, and sex hormone manipulations failed to affect dopaminergic innervation in this area (Kritzer, 2003). These studies clearly emphasize the diversity of the VTA dopaminergic populations in terms of their hormone responsiveness, at least in males. It is important to note, however, that conclusions regarding hormonal responsiveness solely on the expression of ARs or ERs should be drawn with caution because it is now recognized that estrogens and androgens can act rapidly at membrane receptors which may be distinct from their classical intracellular nuclear receptors (Srivastava et al., 2013). Equally, hormones could exert their effects indirectly via non-dopaminergic systems involved in dopaminergic regulation.

Fewer studies have addressed hormonal influences specifically on the mesocortical system in females. Retrograde labeling studies have reported that the distribution of AR in VTA dopaminergic neurons projecting to the PFC in females is similar to that found in males. In contrast, the non-dopaminergic cells in the VTA which project to the PFC were found to be ERβ-positive and ERα-negative in males, but ERβ-negative and ERα-positive in females (Kritzer and Creutz, 2008). The implications of these sex differences require further investigation, but they indicate likely sex-specific effects of estradiol. Estradiol has been shown to affect activity in the mesocortical projections in female rats (McEwen and Alves, 1999), but it remains to be determined whether these differ from effects in males.

### Sex hormone influences during development

When the endogenous gonadal hormone environment is equalized in adult male and female rodents by gonadectomy, there are numerous examples where the effects of treatment with gonadal steroids (especially estrogens) on learning, memory and their structural, electrophysiological and neurochemical correlates, are not the same in males and females (reviewed in (Gillies and McArthur, 2010b)). As discussed under Section ‘Sex hormone influences in adulthood’, this is exemplified in gonadectomized rats by the responsiveness of the female, but not male, mesolimbic system to estradiol. Collectively this body of evidence suggests a fundamental sex dimorphism in the underlying circuitry. Many studies have demonstrated that testosterone exposure of neonatal rat pups is a major driving force for brain masculinization not only in the hypothalamus, but also in other regions important for learning and memory, such as the hippocampus, amygdala and bed nucleus of the stria terminalis (Bangasser and Shors, 2007, Bangasser and Shors, 2008, McCarthy et al., 2008, Arnold, 2009). In line with this hypothesis, one study reported that perinatal exposure to testosterone in male rats is required to achieve normal levels of DA in the frontal cortex at postnatal day 10 (Stewart and Rajabi, 1994). However, whether the perinatal testosterone surge may also be a factor for sexual differentiation of the adult VTA has yet to be fully investigated (Becker, 2009).

As views on the hormonal influences on brain sex differentiation progress, early puberty is emerging as another critical window when rising levels of sex hormones may exert organizational influences on neural pathways which have yet to complete their development (Sisk and Zehr, 2005, Ahmed et al., 2008). The pubertal rise in estrogens in females and testosterone in males therefore provides further possibilities for imprinting brain sex dimorphisms. The mesocortical dopaminergic system most notably continues to develop into young adulthood (Spear, 2000), but detailed knowledge of adolescent sexual differentiation of this pathway is lacking. However, some key observations indicate that puberty is an important time when ovarian steroids contribute to feminization of midbrain dopaminergic circuitry (Becker, 2009). For example, the greater open-field activity displayed by female rats requires exposure to ovarian hormones at the time when gonadal steroidogenesis is first activated transiently in females (around 2 weeks of age) or at the initiation of puberty (postnatal days 30–40), whereas open-field activity may be suppressed (masculinized) by perinatal hormonal exposure (Stewart and Cygan, 1980). As adolescence is a time when sex-specific changes occur in behaviors known to involve dopaminergic systems, including mood, emotional responses, aggression and risk-taking, (Sisk and Zehr, 2005), a better understanding of hormonal influences should be a priority.

### Genomic influences

The perinatal testosterone surge in rats and mice begins around gestational day 17, 4–5 days before parturition. Interestingly, primary mesencephalic cultures derived from rats or mice at embryonic day 13, prior to significant changes in circulating testosterone levels, develop sex-specific characteristics in the absence of sex steroid hormones, as determined by the number of cells expressing tyrosine hydroxylase (the rate-limiting step in DA synthesis indicative of dopaminergic neurons), DA levels in the culture medium and [^3^H]DA uptake (a measure of DAT activity in dopaminergic nerve terminals) (Sibug et al., 1996). These and other observations demonstrate that factors other than sex steroid hormones contribute to sexual differentiation of the brain, and midbrain dopaminergic neurons in particular. Several lines of evidence suggest that the sex chromosomes themselves contribute a genetic component in addition to the epigenetic (hormonal) component to engender biological sex differences (Arnold, 2009). Of note, expression of the SRY gene (the sex determining region of the Y chromosome), which was thought to have a developmentally restricted role in sex determination by directing formation of the testes, has now been identified in a number of adult male non-reproductive tissues including the brain in humans (Ngun et al., 2011), rats and mice (Mayer et al., 2000, Dewing et al., 2006). Furthermore, the SRY protein is expressed in VTA and SNc neurons in post-mortem human and rat brain specimens, where it co-localizes with a subset of neurons expressing tyrosine hydroxylase (Dewing et al., 2006, Czech et al., 2012). SRY has also been shown to positively regulate the expression of enzymes involved in DA synthesis (Milsted et al., 2004, Czech et al., 2012) and silencing *SRY* mRNA reduced DA neuron number in the rodent male SNc, which also compromised SNc (motor) function (Dewing et al., 2006). It remains to be determined whether SRY contributes to sex differences in VTA structure and function. Interestingly, however, investigations using the ‘four core genotype’ mouse model, where genetic sex and gonadal phenotype can be separated (De Vries et al., 2002), found that XX mice (genetic females) showed faster food-reinforced instrumental habit formation than XY mice (genetic males). Moreover, this result occurred regardless of the associated hormonal environment (ovarian or testicular gonadal phenotype) or expression of the *SRY* gene (Quinn et al., 2007). These findings raise the possibility that the sex chromosome complement other than *SRY* may also be influencing sex differences in brain function, including DA-dependent habit-driven behavior. This could be due to direct effects of the Y chromosome genes, to incomplete silencing of X chromosome genes in females, or to sex differences in the genomic imprinting of X-chromosome genes (Arnold and Burgoyne, 2004).

In summary, a full understanding of the developmental mechanisms which underpin sex differentiation of the mesolimbic and mesocortical dopaminergic pathways is notably lacking. This knowledge does, however, have translational relevance for brain disorders involving midbrain dopaminergic systems where their etiology involves a neurodevelopmental component as well as a sex bias (see Section ‘Sex differences in dopaminergic malfunction: impact of stress and neurobiological programing’).

## Sex differences in dopaminergic malfunction: impact of stress and neurobiological programing

Malfunction of the midbrain dopaminergic systems underpins many neurological and psychiatric disorders, including schizophrenia (Lewis and Levitt, 2002), attention deficit/hyperactivity disorder (Solanto, 2002), autism and autism spectrum disorders (Anderson et al., 2008), substance abuse (Melichar et al., 2001), anxiety and depression (Dunlop and Nemeroff, 2007). All of these conditions are characterized by substantial sex differences in their prevalence and/or nature (Ngun et al., 2011, McCarthy et al., 2012). In schizophrenia, for example, the age at onset occurs several years earlier, the risk may be greater, the pathological symptoms may be more severe, and the prognosis may be poorer in men compared with women (Jablensky, 2000, Aleman et al., 2003). For autism there is a much higher male:female ratio in its prevalence (Baron-Cohen et al., 2005). Sex differences are observed in all phases of drug abuse, with women escalating more rapidly to addiction, and being more likely to relapse following abstinence (Carroll et al., 2004, Lynch, 2006, Becker and Hu, 2008). Anxiety disorders and depression are also widely reported to be more prevalent in women (Seeman, 1997). These differences highlight the need to understand the mechanisms which underlie such sex bias in disease which will provide insight into the development of new therapies to meet the specific needs for men and women (Solomon and Herman, 2009).

In all cases, neurobiological factors appear to play an important role in driving sex bias in brain disorders (Kaminsky et al., 2006, Gabory et al., 2009, Lai et al., 2013). The interaction of hormonal and genetic factors during development and/or adulthood, to create sex dimorphisms in normal brain circuitries regulating behavior (Section ‘Mechanisms underlying sexual differentiation of the VTA dopaminergic systems’) may contribute to differential susceptibilities to malfunction. Another factor is environment, with a particular emphasis on the effects of chronic stress, which, as an established risk factor for developing neurological and psychiatric disease (McEwen, 1998, McEwen, 2009, Sapolsky, 2005), may differentially affect males and females (Goel and Bale, 2009). Indeed, a recent review proposed that sex-specific environmental influences should be factored into our current model of brain sex differentiation, along with hormonal and genomic influences (McCarthy and Arnold, 2011). In the context of sex bias in VTA-related disorders, it is therefore important to understand the impact of stress upon the mesolimbic dopaminergic systems.

### The VTA as a target for stress in adulthood

The physiological stress response is typically thought of as an activation of the sympathetic nervous system in order to mount immediate effects on metabolic, cardiovascular and immune systems; this is closely followed by activation of the hypothalamo–pituitary–adrenal (HPA) axis to increase circulating levels of glucocorticoids (cortisol in humans; corticosterone in rodents) (Buckingham, 2006). These glucocorticoid hormones support and prolong physiological stress responses, which are protective, enabling the individual to survive the stressor. Ultimately, the raised glucocorticoid levels exert a negative feedback on the brain and anterior pituitary gland to limit or resolve the body’s reaction to stress and prevent exaggerated or prolonged activation of the HPA axis, which, if not curtailed, become maladaptive and potentially pre-dispose to disease (McEwen, 1998, Sapolsky et al., 2000, de Kloet et al., 2005, McEwen and Gianaros, 2010). Less widely acclaimed, but nonetheless critical for behavioral responses to stress, is activation of the VTA dopaminergic neurons and stimulation of the process of learning and memory by aversive events (Thierry et al., 1976, Roth et al., 1988, Abercrombie et al., 1989, Trainor, 2011). These pathways are better known for their perception of rewarding rather than aversive stimuli, but this function is likely to be distinct from, but co-operative with, those which perceive non-rewarding events (Brischoux et al., 2009, Bromberg-Martin et al., 2010, Roeper, 2013), and are likely to involve a distinct subset of dopaminergic neurons projecting to the amygdala (Guarraci et al., 2000, Sapolsky, 2009). Hence, the VTA dopaminergic circuitry plays a key role in enabling the storage and recall of stressful events, which can then be matched with appropriate behaviors when stressors are encountered subsequently. Such higher cognitive functions are, therefore, key components in enabling the individual to develop stress-coping strategies.

Notably, in humans and experimental species, marked sex dimorphisms are seen in stress sensitivity, as well as in physiological and behavioral responses to stress (Bowman, 2005, Kudielka and Kirschbaum, 2005, Luine et al., 2007, Goel and Bale, 2009, Gillies and McArthur, 2010b, McEwen, 2010), which are characterized typically as ‘fright, flight or fight’ in males, but ‘tend and befriend’ in females (Taylor et al., 2000). Stress also induces sex- and region-specific patterns of structural remodeling in the brain, which are correlated with effects on cognitive and emotional function. For example, in male rats acute stress has been reported to increase dendritic branching and spine synapses in the hippocampus, but to decrease these parameters in the prefrontal cortex, while enhancing vigilance and learning, commensurate with survival in the wild (Leuner and Shors, 2013). Acute stress in females produced opposite responses: dendritic branching and spine synapses were reduced in the hippocampus, but increased in the prefrontal cortex in parallel with withdrawal behavior (commensurate with retreat to safety in the wild) and detrimental effects on learning and memory (Leuner and Shors, 2013). Investigations which specifically focus on the male VTA systems have documented how stress and the glucocorticoid stress hormones impact on the adult mesolimbic and mesocortical dopaminergic systems at behavioral, biochemical, neurochemical and molecular levels (Piazza and Le Moal, 1996, Lindley et al., 1999, Krishnan et al., 2007, Krishnan et al., 2008, Trainor, 2011, Cabib and Puglisi-Allegra, 2012, Lemos et al., 2012, Barik et al., 2013, Niwa et al., 2013, Roeper, 2013). A simple summary of the findings of such studies is complicated by the complexity of VTA dopaminergic responses stress, which vary according to the nature of the stressor (acute versus chronic; physical, psychological or social; repeated homotypic vs. variable) and whether effects on the mesocortical or mesolimbic pathways were investigated. Not surprisingly, therefore, the literature contains apparent contradictions as to whether stressors activate or suppress dopaminergic activity. However, some recent elegant studies in male mice have shed important light on the key role played by dopaminergic neurons in the VTA in stress vulnerability. Based on the premise that stressful events adversely affect behaviors and cause pathological change in the human and rodent brain only in a sub-population of individuals, this work identified unique molecular signatures within the mouse mesolimbic dopaminergic circuit which were specifically associated with either vulnerability or susceptibility using a social defeat paradigm which models depressive-like behavior (Krishnan et al., 2007, Krishnan et al., 2008). These data highlight potential DA-dependent mechanisms in males which could underpin an individual’s susceptibility to succumb to disease. Although parallel studies have not been performed in females, some behavioral responses indicate that the effects of stress on mesocorticolimbic dopaminergic pathways are sexually dimorphic. For example, stress may increase the sensitivity of certain addictive behaviors in females but not males (McCormick et al., 2005); social isolation stress may be anxiogenic in females, but anxiolytic in males (Trainor, 2011); chronic stress impaired male performance in visual and spatial memory tasks, whereas female performance was unaffected or even enhanced (Bowman et al., 2003). Additionally, various types of stress have been shown to alter dopaminergic activity in the mPFC and striatum in one sex but not the other (Bowman et al., 2003, Dalla et al., 2008). In view of the impact which such differences could have on our understanding of disease susceptibility, more research in females and male/female diversity should be a priority (Beery and Zucker, 2011).

### Neurobiological programing of developing VTA dopaminergic systems

It is increasingly recognized that, in many mammalian species, early life experience influences an individual’s sensitivity to stress as well as the pre-disposition to develop mental disorders in later life (Barker, 1995, Heim and Nemeroff, 2002, Lupien et al., 2009, McEwen, 2010, Bale et al., 2010). In humans, for example, retrospective studies and, more recently, prospective studies, document the increased occurrence of emotional problems in children and adolescents whose mothers experienced emotional stress during pregnancy (Talge et al., 2007). Exposure to various types of stressors *in utero*, such as obstetric complications, psychological stress, natural disasters or intra-uterine infections, has also been associated with an increased risk of developing anxiety, depressive states, schizophrenia, ADHD, autism and substance abuse (Melichar et al., 2001, Koenig et al., 2002, Ben Amor et al., 2005, Phillips et al., 2005, Szpir, 2006, Khashan et al., 2008, Weinstock, 2011). These commonest brain disorders typically exhibit a neurodevelopment component and a sex bias, as well as an involvement of midbrain dopaminergic circuitry. Collectively, these observations support the hypothesis that the developing VTA dopaminergic systems have a particular susceptibility to neurobiological programing by early environmental challenge, which differentially impacts male and female brains, thereby leading to sexual dimorphism in susceptibility to DA-associated brain disorders. This is in accord with reports that biological programing of the brain and other structures by gestational exposure to stress/glucocorticoids is sex-specific (Seckl and Holmes, 2007). Further support is offered by a clinical study involving positron emission tomography, which demonstrated that mesolimbic dopaminergic activity was altered in a group of young women, not men, who had experienced early life adversity, compared with those who did not report such experiences (Pruessner et al., 2004). It was proposed that such changes could potentially contribute to the greater susceptibility to developing depressive conditions which is found in this group of females (Vythilingam et al., 2002, Heim and Binder, 2012).

Susceptibility of the developing VTA dopaminergic systems to disruption by adverse environments *in utero* is directly supported by studies employing a variety of animal models of perinatal stress, including maternal exposure to restraint (psychogenic) stress, hypoxia, immune challenge and malnutrition, which alter dopaminergic activity and VTA-dependent functions in the adult offspring, including learning, locomotor activity and addictive behaviors (Henry et al., 1995, Boksa and El-Khodor, 2003, Kippin et al., 2008, Meyer and Feldon, 2009, Gatzke-Kopp, 2010, Rodrigues et al., 2011). These investigations have largely been carried out in male rats or mice, despite numerous reports that early-life programing of metabolic, endocrine, and immune systems, as well as brain function and behavior, is sexually dimorphic in these species (Bowman et al., 2004, Luine et al., 2007, Seckl and Holmes, 2007, Schwarz and Bilbo, 2012). However, sex differences in VTA programing have been proposed on the basis that gestational stress was found to be a sex-specific risk factor for different aspects of substance abuse in pre-clinical models (Thomas et al., 2009). The animal data therefore provide some support for the view that environmental perturbations *in utero* may differentially affect the developmental trajectories in the male and female VTA, leading to a sex bias in the tendency to malfunction in later life.

**Fig. 4:**
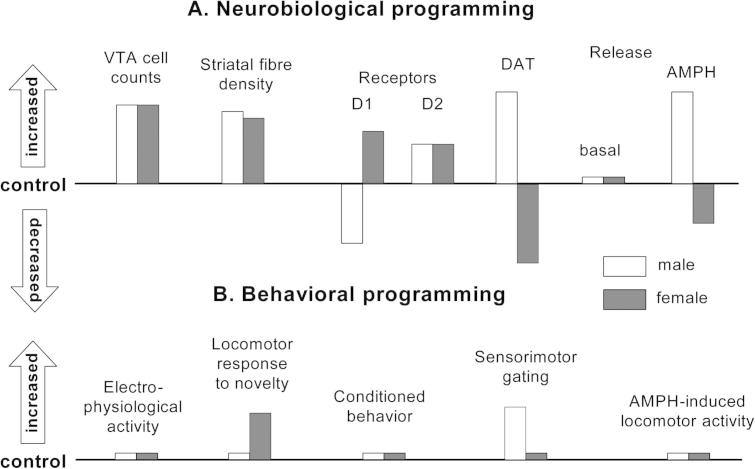
Schematic summary of the directional change in neurobiological and behavioral indicators of VTA dopaminergic activity in adult male and female rats after antenatal glucocorticoid treatment (AGT). Male and female rats exposed to AGT (dexamethasone, 0.5 μg/ml, in drinking water on gestational days 16–19) and controls (dams received normal drinking water) were tested in adulthood. Neurobiological programing (A): VTA Dopaminergic cell counts and striatal fiber density (NAc core and shell) were assessed after immunostaining for tyrosine hydroxylase. Expression levels of DA receptors (D1, D2) and DAT (NAc core and shell) were assessed using autoradiography. DA efflux was measured as described in Fig. 1. Behavioral programing (B): Electrophysiological measurements *in vivo* included extracellular recordings from individual putative DA neurons in the VTA of adult male rats and assessments of spike width, firing rate, inter-spike intervals and percentage of action potentials in spike bursts. Tests of conditioned behavior involved Pavlovian learning in response to appetitive cues predictive of food (autoshaping) and cocaine self-administration using a fixed schedule of reinforcement with ascending doses of cocaine. Sensorimotor gating was tested by analyzing efficacy of a weak sensory stimulus to inhibit a reflexive motor response to a subsequent intense sensory event (pre-pulse inhibition of acoustic startle). Locomotor activity induced by intra-peritoneal injections of amphetamine (0, 0.2, 0.4, 0.8 and 1.2 mg/kg) was recorded using photocell chambers. For full details see Virdee et al. (2013).

An understanding of the mechanisms whereby early environmental insults have enduring effects on the normal functioning of the VTA dopaminergic systems is clearly of paramount importance. A critical common factor in all stress responses is the release of glucocorticoid hormones by the maternal/fetal HPA axis, which are thought to be key players in neurobiological programing in humans and experimental species (Owen et al., 2005, Van den Bergh et al., 2005, Seckl and Holmes, 2007, Davis and Sandman, 2010). Our own work has demonstrated that the rodent VTA systems are direct targets for neurobiological programing by brief exposure to the synthetic glucocorticoid, dexamethasone, toward the end of gestation, with some similarities, but also notable differences, between males and females. Specifically, antenatal glucocorticoid treatment (AGT) increased the adult numbers of dopaminergic neurons in the VTA (by ∼50%) in both sexes (Fig. 2 A, D, F), and this was accompanied by an increase (by ∼40%) in dopaminergic innervation throughout the ventral striatum (NAc core and shell), as well as the dorsal striatum (caudate, putamen) (McArthur et al., 2005, McArthur et al., 2007a) (Fig. 4). Increased neuronal survival did not, however, lead to an increase in basal extracellular DA levels in the NAc in either sex. This could be explained by the marked increase in striatal levels of D2 receptors in AGT-exposed animals (Fig. 4A), which paralleled the increase in terminal density and may represent an adaptation at the level of pre-synaptic auto-inhibitory (D2) receptors to stabilize baseline activity in the face of an AGT-expanded population. Despite these similarities in males and females, AGT-induced changes in other synaptic markers of dopaminergic transmission were profoundly sexually dimorphic (Fig 4). For example, the DAT protein is another presynaptic marker, often taken as an indicator of terminal density, which is a key regulator of synaptic levels of DA. DAT binding density in the NAc (core and shell), as well as the dorsal striatum (caudate/putamen), was similar in control male and female rats, but in AGT-exposed animals its levels were dramatically increased in line with terminal density in males (by 62–140%, depending on region), but decreased (by ∼80%) in females (Fig. 4A) (Virdee et al., 2013). Consequently, DAT levels differed by an order of magnitude in males and females as a result of AGT. Although, surprisingly, this did not affect baseline extracellular levels, AGT dramatically enhanced *in vivo* amphetamine-stimulated DA efflux in males, but reduced it in females (Fig. 1, Fig. 4) (Virdee et al., 2013). As the DA releasing effects of amphetamine arise primarily from its ability to bind to DAT (Fleckenstein et al., 2007), and are commonly regarded as a primary indicator of DA tone, these findings suggest that AGT profoundly alters mesolimbic activity in a sexually dimorphic manner (increased in males, decreased in females).

Behavioral studies provide some support of this hypothesis. For example, in male, but not female, progeny AGT exaggerated the ability of prepulses to inhibit the startle reflex (Virdee et al., 2013), which is indicative of enhanced pre-attentional processing (Swerdlow et al., 2003). As the startle stimulus prevents the fall in NAc DA levels associated with the startle in male rats (Humby et al., 1996), this finding is compatible with the AGT-induced enhancement of mesolimbic dopaminergic activity in males, but not females (Virdee et al., 2013). In contrast, locomotor response to novelty (an indicator of motivational arousal dependent on mesolimbic and mesostriatal dopaminergic systems (Jones and Robbins, 1992)) was attenuated by AGT in females (compatible with the AGT-induced reduction of mesolimbic dopaminergic activity), but not males. However, despite the significant and opposite effect of AGT in males and females to alter amphetamine-stimulated DA efflux in the NAc (Figs. 1 and 4A), amphetamine-induced locomotor activity was remarkably unaffected by AGT in both sexes (Fig. 4B) (Virdee et al., 2013). This unexpected result may be explained by our finding that AGT up-regulated striatal post-synaptic D1 receptors in females (Fig 4A) (Virdee et al., 2013), which may serve to preserve excitability in D1-expressing GABAergic medium spiny output neurons (MSNs) (Lobo and Nestler, 2011) in the face of reduced mesolimbic activity and, hence, maintain basal ganglia output within normal limits. In stark contrast, AGT down-regulated striatal post-synaptic D1 receptors in males (Fig. 4A) (Virdee et al., 2013), which, along with the concurrent increased mesolimbic activity, could serve to normalize basal ganglia output. A similar argument may explain why the substantial AGT-induced neurobiological changes in the mesolimbic dopaminergic system also failed to affect other behaviors, including cocaine self-administration and learning in response to appetitive cues (Fig. 4B) (Virdee et al., 2013), which are known to be reliant on mesolimbic dopaminergic transmission (Jones and Robbins, 1992, Campbell et al., 1997, Parkinson et al., 2002, Dalley et al., 2005). Collectively, these findings demonstrate that apparent behavioral normality in animals exposed to glucocorticoids *in utero* is achieved by AGT-induced adaptive mechanisms in the VTA circuitry which are different, often opponent, in males and females.

In our studies we have used a dose of dexamethasone (∼0.075 mg/kg/day) (McArthur et al., 2006, McArthur et al., 2007b) at or below the clinical dose commonly used in perinatal medicine (∼0.2 mg/kg/day (Ballard and Ballard, 1995). The drug is administered non-invasively via the dam’s drinking water on gestational days 16–19, thereby avoiding any confounding effects of injection stress, and investigations in adult animals were done under basal, non-stressful, conditions which are thought to probe intrinsic functional connectivity (Van den Heuvel and Pasterkamp, 2008). Other studies, using similar levels of dexamethasone exposure *in utero*, corroborate our findings that open-field locomotor activity was lowered in females, but unaffected in males (Kreider et al., 2005), and that behavioral deficits after administration in the drinking water are relatively mild or absent (Hauser et al., 2006, Hauser et al., 2009). In contrast, there are reports in the literature that AGT has more marked long-term effects, including anxiety, depressive-like behavior, drug-seeking behavior and a reduction in mesolimbic dopaminergic transmission in males (Welberg and Seckl, 2001, Oliveira et al., 2006, Rodrigues et al., 2012, Borges et al., 2013). However, these studies have generally employed a higher dose of dexamethasone (1 mg/kg on gestational days 18 and 19 administered subcutaneously) and/or have observed behavioral effects only after either a prolonged exposure to a battery of stressors in adulthood or inherently stressful aspects within the behavioral test. A number of studies have also used pregnant rats supplied by commercial suppliers, which were received into the local animal facility after experiencing transportation stress just a matter of days prior to experimentation (Diaz et al., 1997, Oliveira et al., 2006). The AGT regimen and any association with an additional stressful background at any time-point would, thus, appear to be major factors contributing to contradictions in the literature. The findings do, however, serve to illustrate that neurobiological end-points appear to be more sensitive than behavioral end-points at detecting change. They also demonstrate that behavioral normality is achieved by the midbrain dopaminergic network operating outside its normal limits, and is, therefore, in a state of allostasis, or ‘stability through change’ (McEwen, 1998, Beauchaine et al., 2011). While these AGT-induced adaptations or compensatory mechanisms confer enduring behavioral resilience in certain situations, they may ultimately contribute to the allostatic burden which could represent a pre-disposition to (psych)pathology when further challenged in later life (Brake et al., 1997). As the mechanisms which confer resilience or susceptibility to early environmental challenge occur via sexually dimorphic capacities for molecular adaptations within the VTA dopaminergic systems (Fig. 4), they offer intriguing possibilities for mechanisms which could underpin the sex bias commonly found in midbrain DA-associated disorders.

## Summary

Our understanding of the function of the VTA and, indeed, the brain, derives largely from investigations of the male species. Here, we have reviewed the evidence from both basic science and human data, which indicates notable structural and functional differences in the VTA of females compared with males. The likelihood of a biological basis for sexual diversity in the VTA systems raises important questions regarding sex bias in brain disorders associated with their malfunction. Furthermore, we have highlighted marked sex dimorphisms in the capacity of the VTA systems to adapt or compensate for perturbations in the early-life environment, which are known to increase the risk of malfunction in midbrain dopaminergic pathways and, hence, an individual’s susceptibility to develop psychopathologies in later life. In particular, we have identified sex-specific mechanisms of glucocorticoid neurobiological programing in the mesolimbic DA systems that differentially affect specific male and female behaviors. These relate to female behaviors (motivational arousal), which are altered in depression (more prevalent in women (Kessler, 2003)) and male behaviors (pre-attentional processing/PPI), which are affected in male, but not female, schizophrenic subjects (Kumari et al., 2008). These observations add to the evidence that susceptibility of the relevant circuitry to environmental challenge is sexually dimorphic in a psychopathological context (Bale, 2006). Elucidation of the mechanisms promoting these dimorphisms is an important future challenge and may shed light on sex difference in disease mechanisms and, hence novel sex-specific therapeutic targets. The data also highlight how the midbrain dopaminergic (DA-ergic) systems and the disorders associated with their dysfunction represent excellent prototypes for advancing the field of sex dimorphisms in brain structure, function, and behavior.
